# Morphology Dependent Flow Stress in Nickel-Based Superalloys in the Multi-Scale Crystal Plasticity Framework

**Published:** 2017

**Authors:** Shahriyar Keshavarz, Zara Molaeinia, Andrew C. E. Reid, Stephen A. Langer

**Affiliations:** 1 National Institute of Standards and Technology/Theiss Research, 7411 Eads Ave, La Jolla, CA 92037, USA; 2 Department of Materials Engineering, Purdue University, 701 West Stadium Avenue, West Lafayette, IN 47907, USA; 3 National Institute of Standards and Technology, 100 Bureau Dr, Gaithersburg, MD 20899, USA

**Keywords:** flow stress, morphology, Ni-based superalloys, homogenization, crystal plasticity

## Abstract

This paper develops a framework to obtain the flow stress of nickel-based superalloys as a function of *γ*-*γ′* morphology. The yield strength is a major factor in the design of these alloys. This work provides additional effects of *γ′* morphology in the design scope that has been adopted for the model developed by authors. In general, the two-phase *γ*-*γ′* morphology in nickel-based superalloys can be divided into three variables including *γ′* shape, *γ′* volume fraction and *γ′* size in the sub-grain microstructure. In order tfo obtain the flow stress, non-Schmid crystal plasticity constitutive models at two length scales are employed and bridged through a homogenized multi-scale framework. The multi-scale framework includes two sub-grain and homogenized grain scales. For the sub-grain scale, a size-dependent, dislocation-density-based finite element model (FEM) of the representative volume element (RVE) with explicit depiction of the *γ*-*γ′* morphology is developed as a building block for the homogenization. For the next scale, an activation-energy-based crystal plasticity model is developed for the homogenized single crystal of Ni-based superalloys. The constitutive models address the thermo-mechanical behavior of nickel-based superalloys for a large temperature range and include orientation dependencies and tension-compression asymmetry. This homogenized model is used to obtain the morphology dependence on the flow stress in nickel-based superalloys and can significantly expedite crystal plasticity FE simulations in polycrystalline microstructures, as well as higher scale FE models in order to cast and design superalloys.

## 1. Introduction

**Figure 1.**
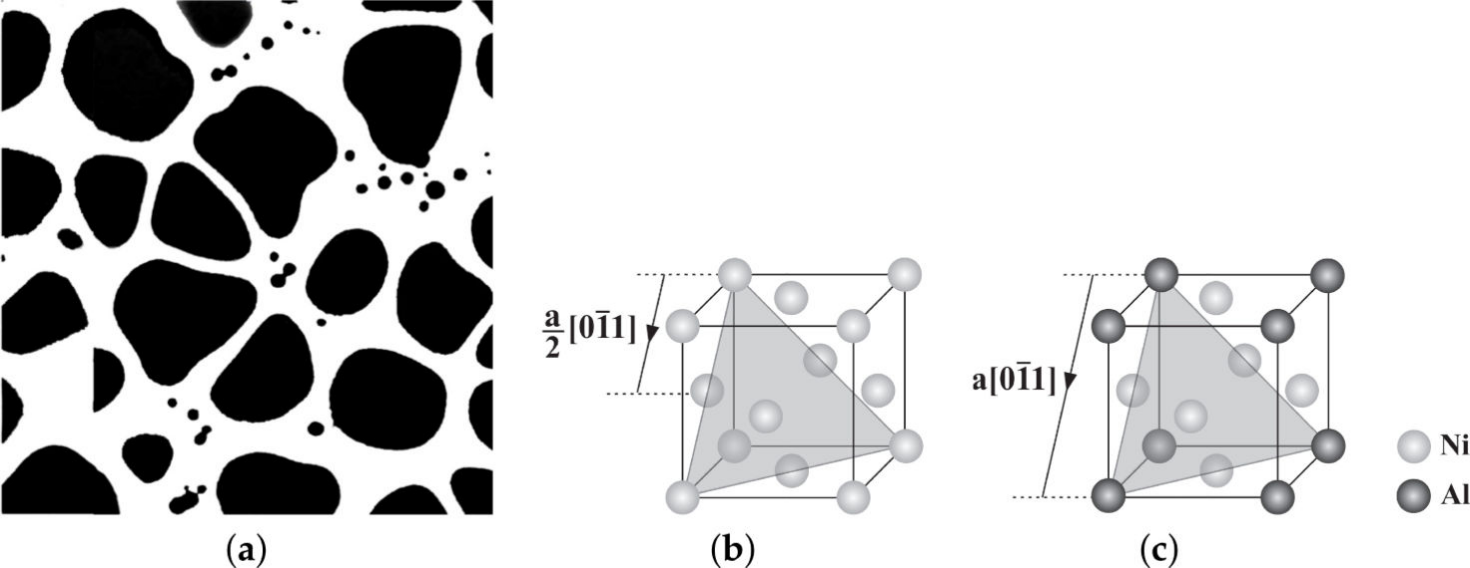
Two-phase nickel superalloys: (**a**) morphology of the two-phase *γ*-*γ′* sub-grain microstructure of Rene 88[5]; (**b**) crystal structure of the *γ* phase; and (**c**) crystal structure of the *γ′* phase.

Some of the major materials used in turbine engines are nickel-based superalloys. There has been enormous investments in improving nickel superalloys in order to reach the desired mechanical properties. To make this process efficient, computational tools are needed to predict the mechanical behaviors of these alloys with consequences for the microstructural design. Flow stress is one of the main aspects of designing these alloys, which has impacts on the other mechanical properties of these alloys, such as fatigue and creep responses. The study is mainly focused on the behavior of single crystals of these alloys in service duties where the morphology of the microstructures can significantly change the mechanical behavior of these materials [2,3]. The morphology of the two-phase nickel superalloys is directly connected to the heat treatment processes. Different heat treatments [4] result in different *γ*-*γ′* arrangements including different shapes, volume fractions and sizes of precipitates. In general, these alloys have a two-phase *γ*-*γ′* microstructure as shown in Figure 1a. The channel *γ* phase (white) is mainly nickel, while the precipitate *γ*-*γ′* phase (black) is an ordered *L*1_2_ crystal structure. The ordered structure *γ′* phase is a strengthening constituent with special thermo-mechanical properties in the overall nickel superalloy. The crystal structures of *γ*-*γ′* are shown in Figure 1b,c. The material in the channel has a regular FCC crystal structure, while in the Ni_3_Al crystal, the corner sites are occupied by the minority (Al) atoms, and the face-centered sites are occupied by the majority (Ni) atoms. The precipitates act as obstacles to the motion of dislocations, which either loop around or shear the precipitates depending on the temperature and stress level. A full dislocation or super-dislocation in *L*1_2_ crystal is 〈110〉, as opposed to $\frac{1}{2}\langle 110\rangle$ in regular FCC crystals for a full dislocation. There is a big difference in the micro-mechanical deformation mechanisms of Ni_3_Al ordered structure from those of a regular FCC structure. The length of the Burgers vector for a full dislocation in the Ni_3_Al ordered structure is different from a regular FCC structure.

**Figure 2.**
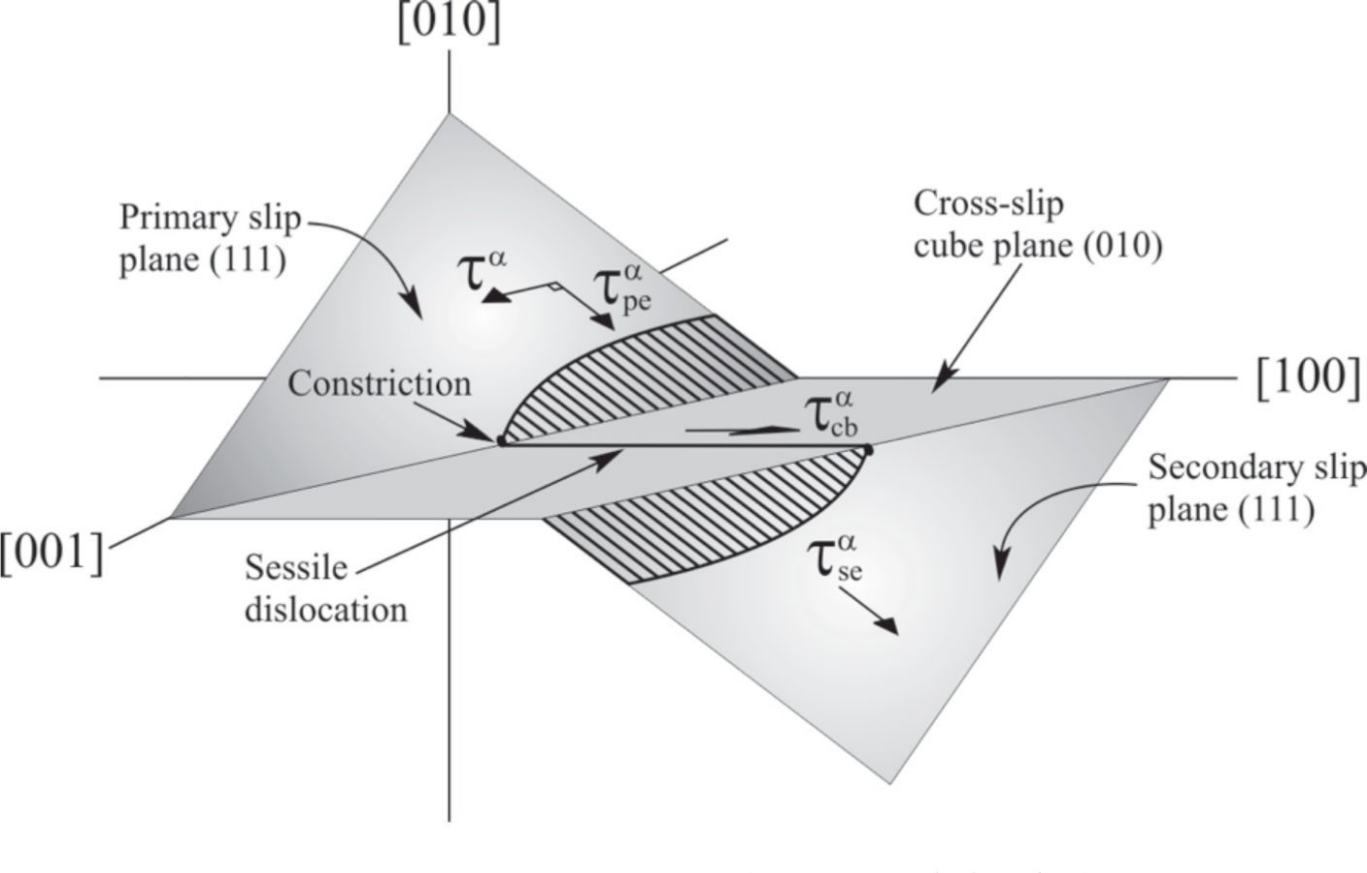
3D configuration of the Kear–Wilsdorf lock.

The mechanical properties, including the dislocation mechanisms under various loading and temperature conditions, have been studied extensively both for single-crystal [6] and polycrystalline [7] Ni-based superalloys. At lower temperatures, octahedral slip systems are mainly active, and slip occurs on these slip systems in both phases. According to the experimental reports, the flow stress in nickel-based superalloys increases when the temperature is elevated up to 1000 K. In this temperature range, most of the dislocations in the intermetallic *γ′* phase become immobile screw dislocations that are locked in a Kear–Wilsdorf (or KW) configuration due to cross-slip [8]. Therefore, they act as barriers for further dislocation motions and result in increasing the flow stress. A 3D configuration of the cross-slip and lock formation is shown in Figure 2. Above 1000 K, cube planes, which are not primary slip systems in FCC materials, can be activated, which has negative impacts on the flow stress; therefore, the flow stress begins to decrease above 1000 K. Above this temperature, edge and screw dislocations on cube planes occur without any cross-slip [1].

The mechanical behavior including creep and fatigue responses must be improved for the next generation of these alloys. This requires studying the lower scales of these materials to get macroscopic scale properties in terms of microstructural data. Hence, it is necessary to incorporate small length scale microstructural mechanisms including dislocation activities. The lower scales in nickel superalloys can be divided into sub-grain and grain scales, where in the sub-grain scale, the study includes the investigation of the dislocation mechanisms by explicit consideration of the *γ*-*γ′* morphology. In the grain scale, there will be a homogenized grain without explicit representation of the *γ*-*γ′* morphology. Therefore, the aim of this work is to develop a morphology-dependent flow stress from the sub-grain scale and bridge the homogenized scale through a multi-scale constitutive model that includes different dislocation activities at different temperatures. The methods yield a significant efficiency advantage, particularly for simulating polycrystals [9–11], since the microstructural RVE problem need not be solved anymore. In general, this multi-scale method can be applied to three scales; however, in this work, we will focus on just the sub-grain and homogenized single-crystal scales.

Crystal plasticity finite element models [12,13] are applied to gather the information hierarchically at each scale to build constitutive models that can be implemented for microstructure-property relations, as well as microstructure design. Meso-scale analyses of superalloys, incorporating precipitate distributions, as well as the grain structure, have been conducted using phenomenological viscoplastic constitutive laws in [14,15]. Hardening parameters in many of the constitutive models have been expressed as assumed functions of the average precipitate size. Analytical models have been proposed using simplifying assumptions for dislocation distributions under uniaxial and monotonic loads in [16]. Crystal plasticity models with implicit dependencies on the grain size and precipitate size and volume fraction have been postulated for a random distribution of precipitates in [17]. Dislocation-density-based hierarchical crystal plasticity models of creep and fatigue have been proposed in [18,19], where the dependence of mechanical properties on microstructural characteristics like average *γ′* precipitate size and volume fraction are accommodated by parameters obtained by fitting with experimental data.

**Figure 3.**
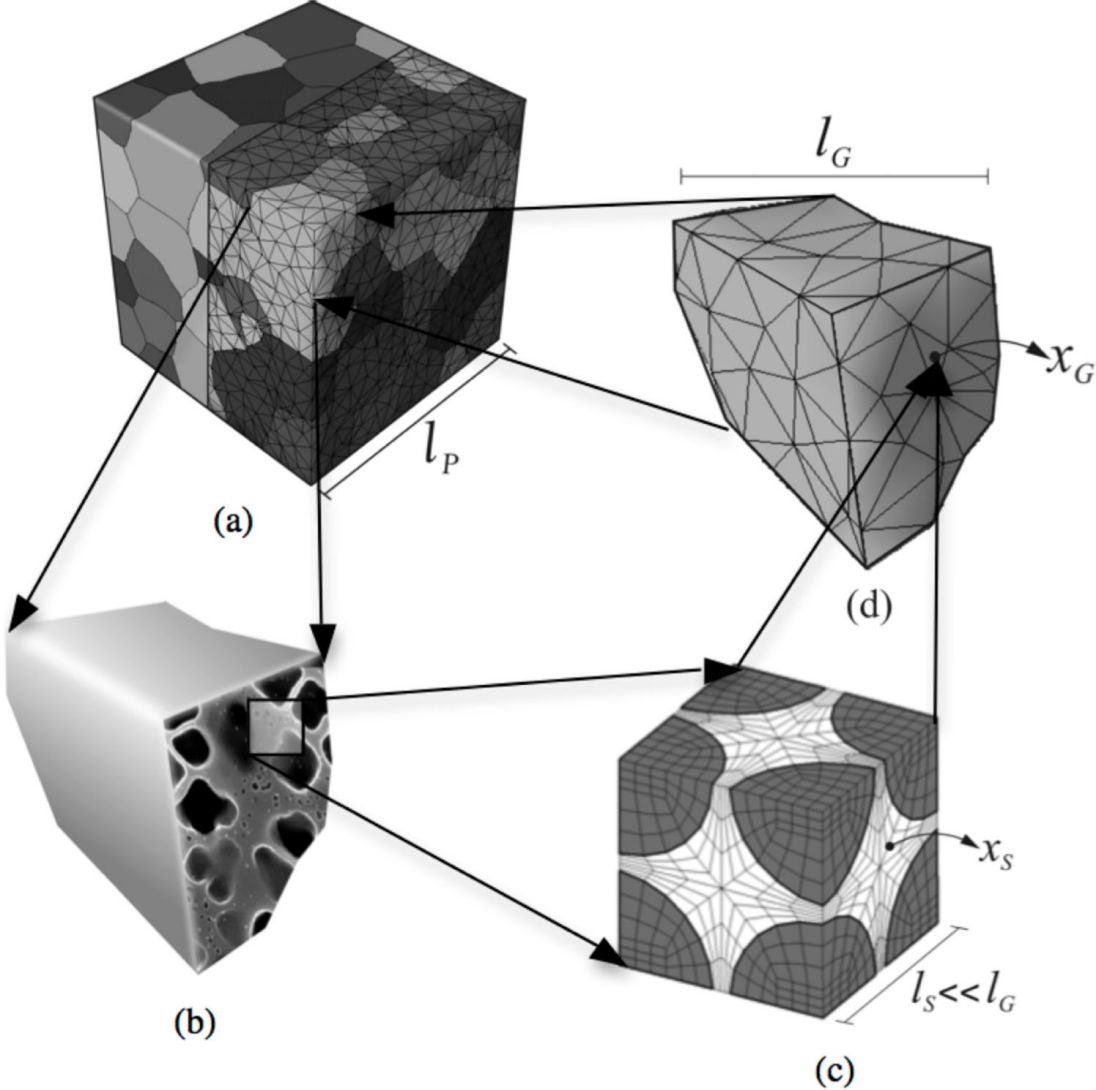
Schematic representation of three scales for Ni-based superalloys in the crystal plasticity finite element framework: (**a**) polycrystalline microstructure showing the grains and CPFEM mesh; (**b**) single grain description with the sub-grain *γ*-*γ′* microstructure; (**c**) discretized sub-grain *γ*-*γ′* microstructural representative volume element (RVE); and (**d**) homogenized crystal plasticity FE model for a single grain.

This paper is aimed at developing a functional form for the flow stress in nickel-based superalloys using a temperature- and orientation-dependent homogenized grain-scale crystal plasticity model with parametric representations of the sub-grain morphology in its evolution laws. The multi-scale approach, which is applied to develop the functional form of the yield stress, is fully presented in [1], and we have adopted that model to provide additional effects of the *γ′* morphology in this manuscript. This multi-scale approach incorporated in the current study, ranging from the sub-grain scale to the meso-scale polycrystalline ensemble, is shown in Figure 3. The cycle starts at the first step with the development of a crystal plasticity finite element or CPFE model of a sub-grain scale representative volume element or RVE, delineating the explicit *γ*-*γ′* morphology. The CPFE model incorporates a non-Schmid size-dependent dislocation density-based crystal plasticity model, in which signed dislocation densities are explicit variables [20,21]. The next step focuses on the homogenized single-crystal scale where an activation-energy-based crystal plasticity model from the homogenization of the dislocation density-based sub-grain model is developed. The homogenized model incorporates temperature variation from room temperature to 1200 K, orientation dependencies to capture asymmetry in tension-compression and activation of cubic slip systems along the effect of the discrete sub-grain morphology through critical morphological parameters. The resulting hierarchical model has the potential of significantly expediting crystal plasticity FE simulations, while retaining accuracy.

Section 2 of this paper introduces the sub-grain scale dislocation density crystal plasticity constitutive laws with anti-phase boundary (APB) shearing of *γ′* precipitates. Section 3 provides a framework for an activation energy-based model at the scale of single crystals. The homogenization procedure that yields morphology-dependent constitutive parameters and their calibration and validation with sub-grain RVE models, as well as experimental results are discussed in Section 4. The morphology dependencies of the mechanical properties in nickel-based superalloys are discussed in Section 5. A summary in Section 6 will conclude the paper.

While this work provides a stand-alone solution to the multi-scale CPFEMproblem it addresses, the model and techniques developed here are also intended to be incorporated into the Object-Oriented Finite Element code, or OOF [22], a general-purpose modeling code intended to assist materials scientists and materials engineers in undertaking computational investigations of structure-property relations in a large variety of systems, including mechanical systems whose behavior is dominated by crystal plasticity.

## 2. Sub-Grain Scale for the *γ*-*γ*_*′*_ Microstructural of Nickel Superalloys

Binary nickel superalloy is a two-phase material consisting of *γ* (Ni) and *γ′* (Ni_3_A1) phases. Plastic deformation is accumulated through crystallographic slip systems, which is different in the two phases, which results in plastic anisotropy in the sub-grain scale. A dislocation-density-based crystal plasticity model, proposed in [2,20,21], is used and implemented to model the rate-dependent plastic behavior. It incorporates the evolution of statistically-stored dislocations (SSDs) in both the *γ* -channels and the precipitates due to various dislocation generation and annihilation mechanisms, while cross-slip dislocations (CSDs) are considered just for *γ′* phase [1]. In order to take into account the gradient of plastic strain at the geometrically-incompatible locations such as the matrix-precipitate interface and grain boundaries, geometrically-necessary dislocations (GND) are also incorporated.

Plastic deformation of nickel-based superalloys can occur by activation of octahedral slip systems and cubic slip systems. However, the dislocation mechanisms are different in the *γ* -channel and in the precipitates. The length of a full dislocation or super-dislocation in precipitates dominant within Ni_3_Al + XX compositions is 〈110〉, almost twice as large as a full dislocation in regular FCC crystals, where a full dislocation is $\frac{1}{2}\langle 110\rangle$. The dominant deformation mechanism in precipitates for almost all ranges of temperatures is the dissociation of a screw super-dislocation into two super-partials, which have a Burgers vector of $\frac{1}{2}\langle 1\bar{1}0\rangle$ and the corresponding creation of a planar fault anti-phase boundary or APB. Afterward, these superpartials basically split into two shockley partials, to bind a complex stacking fault (CSF) having Burgers vectors of $\frac{1}{6}\langle 11\bar{2}\rangle$ as shown in Figure 2. The non-Schmid resolved shear stresses ($\tau_{pe}^{\alpha }$ and $\tau_{se}^{\alpha }$, $\tau_{cb}^{\alpha }$) associated with the shockley partials on primary, secondary octahedral and cube planes, as shown in Figure 2, are included in the constitutive models at both scales. The cross-slip mechanisms in Ni_3_Al + XX compositions do not follow Schmid’s law, commonly employed in crystal plasticity models [23], according to experimental observations. Materials that follow Schmid’s law usually have symmetric evolution for hardness, while the evolution of hardness of the cross-slip mechanism is not symmetric and is different in tension and compression, and it also depends on the crystal orientations. The shear stress on the primary octahedral slip plane $\tau_{pe}^{\alpha }$ constricts the Shockley partials and is partially responsible for the tension-compression asymmetry. For one of the tensile or compressive load direction, $\tau_{pe}^{\alpha }$ constricts the Shockley partials to increase cross-slip rates resulting in the higher flow stress, while in the opposite direction, it hinders cross-slip with a decrease in flow stress.

### 2.1. Crystal Plasticity Model for the Sub-Grain Model

The constitutive model admits a multiplicative decomposition of **F** = **F**^*e*^**F**^*p*^ where the total deformation gradient **F** contains an inelastic, incompressible part **F**^*p*^ associated with just slip without rotation and an elastic part **F**^*e*^ that accounts for rigid-body rotations and elastic stretching. For the plastic velocity gradient **L**^*p*^, the plastic shear strain rate ${\dot{\gamma }}^{\alpha }$ on the slip system *α* (including the slip direction $m_{0}^{\alpha }$ and slip plane normal $n_{0}^{\alpha }$ in the reference configuration) and the Schmid tensor $s_{0}^{\alpha }=m_{0}^{\alpha }⊗n_{0}^{\alpha }$ can be employed to calculate the evolution of plastic deformation as:

(1) $$L^{p}={\dot{F}}^{p}F^{-p}=\sum_{\alpha =1}^{N}{\dot{\gamma }}^{\alpha }s_{0}^{\alpha }=\sum_{\alpha =1}^{N}{\dot{\gamma }}^{\alpha }m_{0}^{k}⊗n_{0}^{\alpha }$$

The stress-strain relation invokes the second Piola–Kirchoff stress **S** and its work conjugate Green–Lagrange strain tensor **E**^*e*^ in the intermediate configuration:

(2) $$S=det\left(F^{e}\right)F^{e^{-1}}\sigma F^{e^{-T}}=C:E^{e}\;and\;E^{e}=\frac{1}{2}\left(F^{e^{T}}F^{e}-I\right)$$

where **C** is a fourth order anisotropic elasticity tensor, ***σ*** is the Cauchy stress tensor and **I** is the identity tensor.

The plastic shear strain rate on a slip system is given by the Orowan equation as ${\dot{\gamma }}^{\alpha }=\rho_{M}^{\alpha }b^{\alpha }v^{\alpha }$ with the mobile dislocation density as $\rho_{M}^{\alpha }$, Burgers vector as *b*^*α*^ and the dislocation velocity as $v^{\alpha }$ for a given slip system. The crystal plasticity framework incorporating the signed dislocation density used for superalloys in [2] is modified in the current study for rate-dependent plastic behavior. The modifications include adding cross-slip dislocation densities, the temperature dependency of cross-slip shear resistance and considering cubic slip systems in addition to octahedral slip systems. In general, the velocity of dislocations can be written as:

(3) $$v^{\alpha }=v_{0}\exp\left(-\frac{Q}{K_{B}\theta }\right)\sinh{\left(\frac{\left|\tau^{\alpha }\right|-\tau_{\text{pass}}^{\alpha }}{\tau_{\text{cut}}^{\alpha }}\right)}^{p}\mathrm{sign}\left(\tau^{\alpha }\right)$$

The initial dislocation velocity is considered as $v_{0}=\lambda_{\alpha }f_{0}$ where *f*_0_ is the attack frequency and *λ*_*α*_ is a temperature-dependent jump width. The jump width *λ*_*α*_ can be calculated in terms of parallel and forest dislocation densities as $\lambda_{\alpha }=\frac{c_{0}}{\sqrt{\rho_{P}^{\alpha }\rho_{F}^{\alpha }}}(\frac{\theta }{\theta_{ref}}{)}^{c_{1}}$. The temperature-dependent velocity in this equation includes the absolute temperature *θ* and the activation energy Q. To provide a control to the velocity for a given hardening evolution, an exponent *p* is introduced in the form of the hyperbolic term in the current study. The dislocation velocity in this equation is a function of resolved shear stress *τ*^*α*^, a component of applied load and slip system resistances parallel to the slip system or passing stress $\tau_{pass}^{\alpha }$ and perpendicular to the slip system or cutting stress $\tau_{cut}^{\alpha }$. The passing stress is the result of the interaction of mobile dislocations with other dislocations and their networks in the slip plane, while the cutting stress is the result of the mobile dislocations cutting the forest dislocations with density $\rho_{F}^{\alpha }$, which are perpendicular to the slip plane. The passing and cutting stresses are [24]:

(4) $$\tau_{\text{pass}}^{\alpha }=c_{2}Gb\sqrt{\rho_{p}^{\alpha }+\rho_{F}^{\alpha }},\;\tau_{\text{cut}}^{\alpha }=\frac{c_{3}K_{B}\theta }{b^{2}}\sqrt{\rho_{F}^{\alpha }}$$

where *G* is the shear modulus and *c*_2_ and *c*_3_ are material constants. Parallel and forest dislocation densities are due to statistically-stored dislocations or SSDs, which account for lock formation, dipole formation, athermal annihilation, thermal annihilation and geometrically-necessary dislocations or GNDs to account for the gradient of the plastic deformation between two phases [24]. Finally, the mobile dislocation density $\rho_{m}^{\alpha }$ can be written as a function of forest and parallel dislocation densities along with the temperature as:

(5) $$\rho_{M}^{\alpha }=\frac{c_{9}K_{B}\theta \sqrt{\rho_{F}^{\alpha }\rho_{P}^{\alpha }}}{Gb^{3}}$$

where *c*_9_ can be evaluated from *c*_2_ and *c*_3_ as $c_{9}=\frac{2c_{3}}{c_{2}}$.

In general, dislocation activities or plastic deformation in the two-phase nickel superalloys begin in the *γ* channel when the resolved shear stress is larger than the slip system resistance or passing stress. Then, the SSDs evolve, and due to gradients in plastic deformation in the *γ* channel and *γ*′ precipitates, the GNDs also evolve; at some point, the dislocation in the channel has enough stress to cut through the precipitates, generating dislocation nucleation in the *γ′* phase. The dislocation nucleation criterion in the *γ′* phase can be divided into two categories, namely: (1) for octahedral slip systems with the non-Schmid effects and (2) for the cubic slip systems without the non-Schmid effects. To accommodate the criterion in the crystal plasticity framework, the APB shearing criterion in [2] is extended as follows:

(6) $$\tau_{eff}^{\alpha }=\left|\tau^{\alpha }\right|-\tau_{pass}^{\alpha }>\tau_{c}$$

where:

(7) $$\tau_{eff}^{\alpha }=\{\begin{array}{ll} \left|\tau^{\alpha }\right|-\tau_{pass}^{\alpha } & for\;\left|\tau^{\alpha }\right|>\tau_{pass}^{\alpha }\\ 0 & for\;\left|\tau^{\alpha }\right|\leq \tau_{pass}^{\alpha_{as}} \end{array}$$

This criterion is valid for both octahedral and cubic slip systems; however, the critical shear stress for octahedral slip systems stated in Equation (7) is a function of three non-Schmid components of the shear stresses on the primary and secondary octahedral slip planes, as well as the cube plane, as well as the anti-phase boundary energy on both octahedral and planes. On the other hand, *τ*_*c*_ for the cubic slip system is just a function of temperature. Overall, the critical shear stress for both octahedral and cubic slip systems can be written as [1]:

(8) $$\tau_{c}^{\alpha }=\{\begin{array}{ll} \tau_{co}^{\alpha }=\tau_{co}^{\alpha }\left(\tau_{pe}^{\alpha },\tau_{se}^{\alpha },\tau_{cb}^{\alpha },\theta ,\Gamma_{111},\Gamma_{010}\right) & \text{on}\;\text{octahedral}\;\text{slip}\;\text{systems}\\ \tau_{cc}=\tau_{cc}(\theta ) & \text{on}\;\text{cube}\;\text{slip}\;\text{systems} \end{array}$$

There are two factors, important in increasing dislocation densities of the cross-slip mechanism, creating thermally-activated constrictions and increasing temperature. The critical shear stress corresponding to Equation (8) evolves by increasing the dislocation densities of the cross-slip mechanism. However, the strength of obstacles decreases with an increase in temperature. Consequently, there is a competition between increasing strength due to the formation of KW locks and obstacle strength reduction with increasing temperature.

### 2.2. Material Constants in the Constitutive Law

There are two types of material constants in Equations (3)–(8). Constants in the first type can be found in the literature [25]. They include *h*, Γ^010^, Γ^111^, *b*, *μ* and *ρ*_0_, which have values of 0.3, 0.083, 0.3, 2.49 × 10^−10^ m, 142.2 GPa and 5.0 × 10^15^ m/m^3^, respectively. The statistically-stored dislocation density needs a proper initial value, which we derive based on the experiments and can be stated as a function of temperature:

$$\rho_{SSD0}=\{\begin{array}{ll} 4.04\times 10^{11}-3.34\times 10^{8}\theta & \theta \leq 659K\\ 2.42\times 10^{11}-0.87\times 10^{8}\theta & 659K<\theta \leq 930K\\ 13.28\times 10^{11}-12.58\times 10^{8}\theta & 930K<\theta \leq 1000K\\ 1.53\times 10^{11}-0.8\times 10^{8}\theta & \theta >1000K \end{array}$$

As discussed, the critical shear stress in Equation (7) for cubic slip systems was just a function of temperature because the cross-slip mechanism only occurs for the octahedral slip systems. From the data given in [1], the cubic slip resistance can be calibrated as:

$$\tau_{cc}=\{\begin{array}{ll} 460\;MPa & \theta \leq 915\text{K}\\ 1558-1.2\theta \;MPa & otherwise \end{array}$$

The elastic stiffness tensor **C**_*αβ*_ = **C**_*βα*_(*α* = 1, …, 6, *β* = 1, …, 6) is considered to have the cubic symmetry for both phases. The elastic stiffness tensor components are functions of temperature. For the *γ* phase, the non-zero components of the stiffness tensor can be derived [26]:

$$C_{11}=C_{22}=C_{33}=(298-0.096\theta )\;GPa\;C_{44}=C_{55}=C_{66}=(139-0.035\theta )\;GPa\;C_{12}=C_{13}=C_{23}=(191-0.057\theta )\;GPa$$

For the *γ′* phase, the non-zero components of the stiffness tensor are:

$$C_{11}=C_{22}=C_{33}=(325-0.096\theta )\;GPa\;C_{44}=C_{55}=C_{66}=(144-0.035\theta )\;GPa\;C_{12}=C_{13}=C_{23}=(209-0.057\theta )\;GPa$$

The rest of the material constants in the constitutive model, corresponding to the second type, are calibrated from experiments on single-crystal CMSX-4 in [1]. The alloy contains a 70% volume fraction of predominantly cuboidal *γ′* precipitates of an average size of 0.45 μm with the average size of the RVE of 0.5 μm.

The second type of material constants are the ones calibrated according to the constitutive model to capture the experimental data. In general, these constants can be divided into three categories: (1) yield state constitutive constants, (2) temperature state material constants; and (3) hardening state material constants. The yield state includes stresses corresponding to the onset of plastic deformation up to 0.2% offset strain. The temperature material constants are responsible for the anomalous behavior of Ni3Al alloys. The hardening material constants reflect the interaction of different dislocation mechanics, which result in hardening after the yield sate. The parameters corresponding to the yield state, temperature state and hardening state are listed in Tables 1–3.

### 2.3. Implementation of the Crystal Plasticity Constitutive Model into to the Code

The crystal plasticity constitutive model explained in Section 2 for the two-phase *γ*-*γ′* is implemented in a crystal plasticity FE (CPFE) code. The rate-dependent constitutive model requires the use of a time-integration scheme; therefore, an implicit time-integration scheme is implemented. In the implicit schemes developed in [25,27], backward Euler time integration methods are used to solve a set of nonlinear equations in the time interval *t* ≤ *τ* ≤ *t* +Δ*t* using iterative Newton–Raphson methods. The algorithm proposed in [27] needs the solution of six equations corresponding to the number of second Piola–Kirchoff stress components, while that in [25] solves equations equal to the number of slip systems (>6 for the FCC systems). The integration algorithm in [27] is adopted in this work, which requires known deformation variables, e.g., **F**(*t*) and **F**^*p*^(*t*), *ρ*_*SSD*_(*t*), *ρ*_*CSD*_(*t*) and *ρ*_*GND*_(*t*) and slip system deformation resistances $\tau_{\text{pass}}^{\alpha }(t)$, $\tau_{\text{cut}}^{\alpha }(t)$, $\tau_{\text{co}}^{\alpha }(t)$ and $\tau_{\text{co}}^{\alpha }(t)$ at time *t*, as well as **F**(*t* + Δ*t*), as inputs to a material update routine CPFEM-MAT. By, integrating Equation (1), the plastic part of the deformation gradient at time *t* +Δ*t* is expressed as:

(9) $$F^{p}(t+\Delta t)=\left(I+\sum_{\alpha =1}^{N}\Delta \gamma^{\alpha }m_{0}^{\alpha }⊗n_{0}^{\alpha }\right)F^{p}(t)=\left(I+\sum_{\alpha =1}^{N}\Delta \gamma^{\alpha }s_{0}^{\alpha }\right)F^{p}(t)$$

By substituting the expressions for **F**^*p*^(*t* + Δ*t*) and **F**(*t* + Δ*t*) into Equations (1) and (9), the incremented second Piola–Kirchoff stress is calculated as:

(10) $$\;S(t+\Delta t)=\frac{1}{2}C:\left(F^{p^{-T}}(t+\Delta t)F^{T}(t+\Delta t)F(t+\Delta t)F^{p^{-1}}(t+\Delta t)-I\right)\;=\frac{1}{2}C:(A(t+\Delta t)-I)-\sum_{\alpha =1}^{N}\Delta \gamma^{\alpha }\left(S(t+\Delta t),\tau_{\text{pass}}^{\alpha }(t),\tau_{\text{cut}}^{\alpha }(t),\tau_{\text{co}}^{\alpha }(t),\tau_{\text{oc}}^{\alpha }(t)\right)C^{\alpha }\;\;=S^{tr}-\sum_{\alpha =1}^{N}\Delta \gamma^{\alpha }\left(S(t+\Delta t),\tau_{\text{pass}}^{\alpha }(t),\tau_{\text{cut}}^{\alpha }(t),\tau_{\text{co}}^{\alpha }(t),\tau_{\text{oc}}^{\alpha }(t)\right){\bar{C}}^{\alpha }$$

where:

(11) $$A(t+\Delta t)=F^{p^{-T}}(t+\Delta t)F^{T}(t+\Delta t)F(t+\Delta t)F^{p^{-1}}(t+\Delta t)\;\text{and}\;{\bar{C}}^{\alpha }=\frac{1}{2}C:\left(As_{0}^{\alpha }+s_{0}^{\alpha T}A\right)$$

A nonlinear Newton–Raphson iterative method is used to find the second Piola–Kirchoff stress stated in Equation (10):

(12) $$S^{i+1}(t+\Delta t)=S^{i}(t+\Delta t)-{\left(I+\sum_{\alpha =1}^{N}C^{\alpha }⊗\frac{\partial \Delta \gamma^{\alpha }}{\partial S^{i}}\right)}^{-1}G^{i}\;\text{where}\;G=S-S^{tr}+\sum_{\alpha =1}^{N}\Delta \gamma^{\alpha }C^{\alpha }$$

The plastic deformation gradient can be calculated by solving this equation. Subsequently, Cauchy stress and the tangent stiffness matrix $W_{ijkl}=\frac{\partial \sigma_{ij}}{\partial \epsilon_{kl}}$ can be computed by having Cauchy stress and strain in CPFEM-MAT and passed on to the FE program for the equilibrium equation. The time integration scheme at the Gauss point level is detailed in Table 4.

### 2.4. Validation of the Sub-Grain CPFEM Model

The results of the crystal plasticity constitutive model developed for the dislocation nucleation in both *γ*-*γ′* phases and stated in Section 2 in the CPFEM framework are compared with experimental data, which were performed by different experts [28–30]. These experiments are carried out on CMSX-4 nickel superalloys or on a very similar compound; therefore, the RVE is constructed for a regular array of cubic precipitates with a 70% precipitate volume fraction. The dimensions of the RVE are 0.5 μm × 0.5 μm × 0.5 μm. The size of cubic *γ* particles allocated symmetrically at the eight corners is 0.45 μm. The CPFE model of the microstructural RVE is discretized into 2200 elements using eight-noded trilinear brick elements. To emulate the experimental conditions, constant strain-rate and creep loads are applied to the top surface, while rigid body modes are suppressed by applying boundary conditions on the opposite bottom surface.

**Figure 4.**
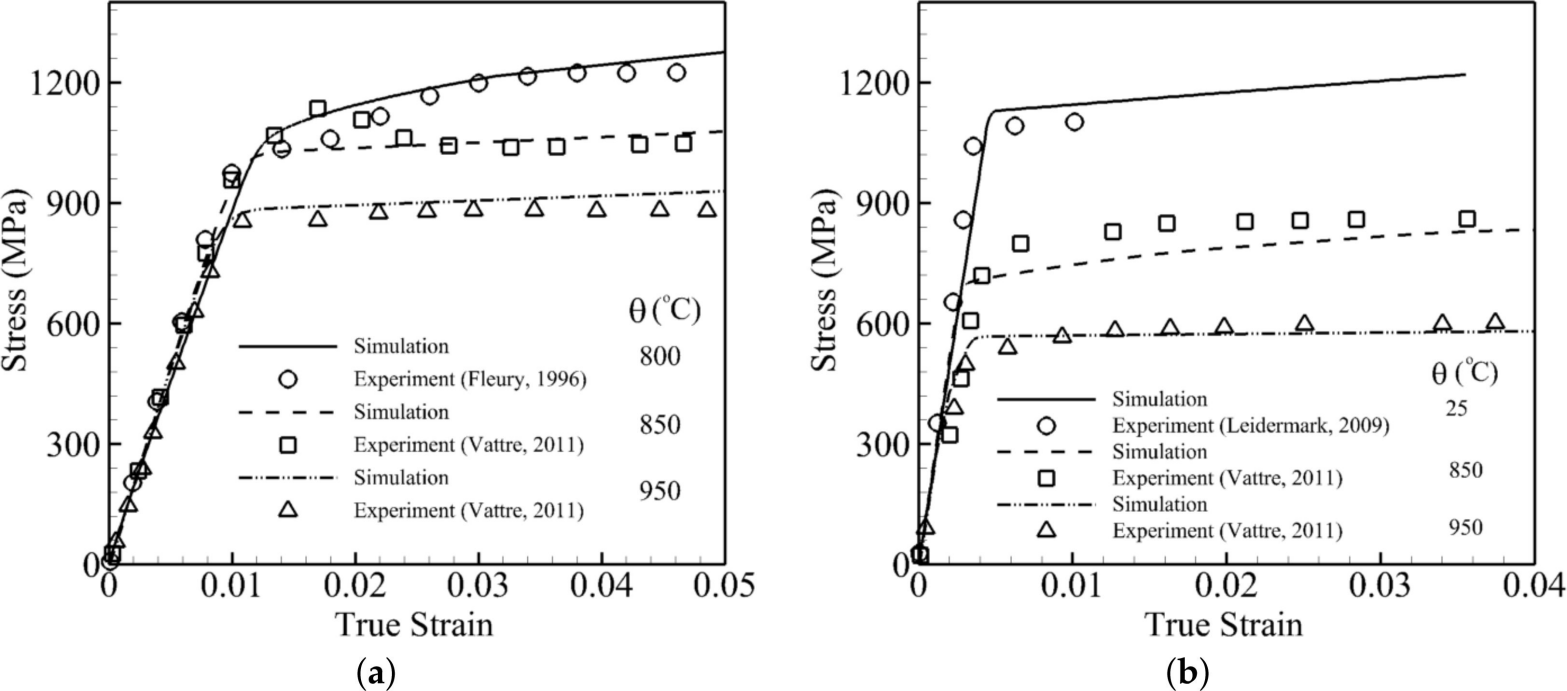
Volume-averaged true stress-logarithmic strain response by CPFEM and experiments [28–30] for different temperatures of a single crystal of nickel-based superalloy (CMSX-4): (**a**) [1] orientation under a tensile constant strain rate of 0.001 s^−1^ (**b**) [111] orientation under a tensile constant strain rate of 0.0001 s ^−1^.

The model is able to predict the mechanical behaviors of nickel-based superalloys for a wide range of temperatures, different orientations and different strain rates in the quasi-static range and exhibits asymmetry in tension and compression. Tensile constant strain tests are performed for four temperatures including room temperature, 800 °C, 850 °C and 950 °C. These two sets of simulations are executed with respect to two [1] and [111] orientations at different temperatures. For orientations close to [1], three constant strain rate simulations, which correspond to the experiments, are performed at 800 °C [28], 850 °C [30] and 950 °C [30]. The tensile constant strain rate is 0.001 s^−1^. The volume-averaged stress-strain responses are subsequently compared with the experimental data in Figure 4a. The simulations show a very good agreement with experimental data. It can be observed that the yield stress and hardening drop as temperature increases. The second set of comparisons is done for an orientation close to [111] where three constant strain rate simulations are performed at 25 °C [29], 850 °C [30] and 950 °C [30]. The constant tensile strain rate is 0.0001 s^−1^. The volume-averaged stress-strain responses are subsequently compared with experimental data in Figure 4b. The simulations show a good agreement with experimental data. It can be observed that the yield stress at high temperature is much less than at room temperature due to the activation of cubic slip systems.

## 3. Grain-Scale Crystal Plasticity Framework

The homogenized single-crystal grain-scale for nickel superalloys proposed in [2,31–33] is employed. The model is almost similar to the sub-grain model where hardening parameters are a function of plastic deformation instead of dislocation densities. The constitutive model incorporates an evolving thermal shear resistance, as well as an athermal shear resistance due to the plastic deformation. For a slip system *α*, the plastic shear strain rate can be calculated from the Orowan equation as:

(13) $${\dot{\gamma }}^{\alpha }=\{\begin{array}{ll} 0 & \text{for}\;\tau_{\text{eff}}^{\alpha }\leq 0\\ {\dot{\gamma }}_{*}^{\alpha }\exp\left\{-\frac{Q}{K_{B}\theta }{\left[1-{\left(\frac{\left|\tau_{\text{eff}}^{\alpha }\right|}{\tau_{\text{cut}}^{\alpha }}\right)}^{p}\right]}^{q}\right\}\mathrm{sign}\left(\tau^{\alpha }\right) & \text{for}\;0<\tau_{\text{eff}}^{\alpha }\leq \tau_{\text{cut}}^{\alpha } \end{array}$$

For the slip system *α*, ${\dot{\gamma }}_{*}^{\alpha }$ is a reference strain-rate as a function of plastic strain and morphological parameters [1]. The temperature-dependent slip system resistance *s*_*α*_ is assumed to be a result of a thermally-activated obstacle to slip $\tau_{cut}^{\alpha }$ or $s_{*}^{\alpha }$ and partly due to the athermal obstacles $\tau_{pass}^{\alpha }$ or $s_{a}^{\alpha }$ as defined in [1]. The driving force for dislocation motion on the slip system *α* is comprised of the difference between the athermal shear resistance and the resolved shear stress.

The athermal shear resistance reflecting the effect of parallel dislocations in the slip direction **m**^*α*^ is defined as ${\dot{s}}_{a}^{\alpha }=\sum_{\beta =1}^{N}h_{a}^{\alpha \beta }\left|{\dot{\gamma }}^{\beta }\sin\left(n^{\alpha },t^{\beta }\right)\right|$ where **n**^*α*^ is the slip-plane normal, **t**^*α*^ = **m**^*α*^ × **n**^*α*^. The thermal shear resistance or cutting stress incorporates two dislocation mechanisms. The first mechanism is employed in order to capture the effect of forest dislocations as ${\dot{s}}_{*}^{\alpha }=\sum_{\beta =1}^{N}h_{*}^{\alpha \beta }\left|{\dot{\gamma }}^{\beta }cos\left(n^{\alpha },t^{\beta }\right)\right|$. The evolution of total shear slip resistance is ${\dot{s}}^{\alpha }=\sqrt{{\left({\dot{s}}_{a}^{\alpha }\right)}^{2}+{\left({\dot{s}}_{*}^{\alpha }\right)}^{2}}$. The interactions between slip systems are taken to be isotropic; in other words, the coefficients are the same, i.e., $h_{a}^{\alpha \beta }=h_{*}^{\alpha \beta }=h^{\alpha \beta }$. Each component of *h*^*αβ*^ is the deformation resistance on slip system *α* due to shearing on slip system *β*. It describes both self and latent hardening as:

(14) $$h^{\alpha \beta }=q^{\alpha \beta }h^{\beta },\;\text{where}\;h^{\beta }=\left[h_{0}{\left(1-\frac{s^{\beta }}{s_{sat}^{\beta }}\right)}^{r}\right]sign\left(1-\frac{s^{\beta }}{s_{sat}^{\beta }}\right)$$

The parameter *h*^*β*^ is the resistance parameter for the dependent self-hardening rate; $s_{sat}^{\beta }$ is the saturation value of reference shear stress; and exponent *r* is a material constant. The parameter $q^{\alpha \beta }=q+(1-q)\delta^{\alpha \beta }$ or the interaction coefficient matrix includes *q* as a latent-hardening parameter and is chosen to be 1.4.

The activation enthalpy for cross-slip is extended in the same approach employed in the sub-grain scale presented in Equation (8) where the rate of cross-slip resistance is a function of the anti-phase boundary energies on the octahedral and cube planes, as well as on the non-Schmid components of the resolved shear stress. The non-Schmid components $\tau_{pe}^{\alpha }$, $\tau_{se}^{\alpha }$ and $\tau_{cb}^{\alpha }$ are considered to have the same duties in the dislocation dissociation and slip on the octahedral slip systems and contribute to their slip resistances. According to [34], the cross-slip shear resistance can be stated as:

(15) $$\tau_{\text{crossco}}^{\alpha }=\xi_{0}\exp\left(\frac{A}{\theta -\theta_{c}}\right)\mu \sqrt{\rho_{0}\exp\left(-\frac{H^{\alpha }}{K_{B}\theta }\right)}\;\;\text{where}\;H^{\alpha }=c_{H}\left\{h+k_{1}\left(t_{\text{pe}}^{\alpha }-k_{1}t_{\text{se}}^{\alpha }\right)+\sqrt{\left(\frac{1}{\sqrt{3}}-\frac{\Gamma^{010}}{\Gamma^{111}}+\left|t_{\text{cb}}^{\alpha }\right|\right)\frac{b}{B}}\right\}$$

The total thermal shear resistance or cutting stress can be calculated as:

(16) $$\tau_{\text{cut}}^{\alpha }=s_{*}^{\alpha }+s_{\text{cross}}^{\alpha }$$

Material parameters in the above homogenized constitutive model are calibrated for the superalloy CMSX-4 single crystals in [2] and listed in Table 5.

## 4. Homogenized Single-Crystal Model from the Sub-Grain RVE Model

The morphology-dependent constitutive parameters in Equations (13) and (14) for the activation-energy-based crystal plasticity model are considered to be governed by the Hill–Mandel principle of macro-micro energy equivalence [35], where the micromechanical analysis is conducted with the sub-grain RVE model. The constitutive model includes functional parameters, which are formulated in terms of critical morphological variables and are fitted by computational homogenization of the sub-grain RVE model response.

### 4.1. Morphological Parameters in the Sub-Grain Microstructural RVE

The sub-grain microstructural RVE consists of *γ′* precipitates homogeneously distributed in a matrix *γ* phase as shown in Figure 3c. The two-phase *γ*-*γ′* microstructure is characterized as three morphology parameters including: (i) the volume fraction of the *γ′* precipitates, (ii) the shape factor *n* of the *γ′* precipitates and (iii) the minimum channel width *l*_*c*_ between the *γ′* precipitates. The volume fraction is defined as the ratio of the *γ′* precipitate volume to the total RVE volume, i.e., $v_{f}=\frac{V_{\gamma^{\prime }}}{V_{RVE}}$. The shape factor of the precipitates is described in terms of the exponent of a superellipsoid: ${\left(\frac{x}{a}\right)}^{n}+{\left(\frac{y}{b}\right)}^{n}+{\left(\frac{z}{c}\right)}^{n}=1$, where *a*, *b* and *c* are the dimensions of the three principal axes and *n* is the shape exponent. Here, *a* = *b* = *c* for equiaxed precipitates. A value *n* = 2 corresponds to spherical precipitates, while *n* → ∞ corresponds to cubic ones. In the homogenization procedure, a transformed shape factor *n*_1_ = *tan*^−1^(*n*) is used to avoid singularity.

### 4.2. Morphology-Dependent Constitutive Parameters in the CP Model

Plastic shear deformation and hardening constitutive parameters in the single crystal grain-scale of AE-CP model are functions of the statistically-stored dislocations and cross-slip dislocation densities. At this scale, the homogenized single-crystal scale, the distribution of the dislocations is uniform. At the sub-grain scale, the distribution is not uniform because in a two-phase material, there will be a gradient in the dislocation densities, which generates geometrically-necessary dislocations. GNDs can change significantly when the morphology of the RVE changes. In other words, when the shape, size and distance between precipitates change, which normally occurs during the heat treatment process, the mechanical response of the RVE will vary. Hence, morphological parameters should also be incorporated into the homogenization process through these functions to consider the gradient of plastic shear strain corresponding to GNDs. Sensitivity analyses in [2] show that the initial thermal shear resistance, the reference slip-rate, the saturation shear stress and the cross-slip shear resistance are functions of the morphology. Thus, in Equations (13) and (14), the parameters $s_{*0}^{\alpha }\left(n_{1},v_{p},l_{c}\right)$, ${\dot{\gamma }}_{*}\left(n_{1},v_{p},l_{c}\right)$, $s_{sat}^{\alpha }\left(n_{1},v_{p},l_{c}\right)$ and $s_{cross}^{\alpha }\left(n_{1},v_{p},l_{c}\right)$ can be derived in terms of morphology, as well as (*γ*^*α*^, ▽*γ*^*α*^).

### 4.3. Functional Forms of the Single-Crystal Homogenized Constitutive Parameters

Four constitutive parameters in the single-crystal grain scale, $s_{*0}^{\alpha }\left(n_{1},v_{p},l_{c}\right)$, $k_{*}\left(n_{1},v_{p},l_{c}\right)$, $k\left(n_{1},v_{p},l_{p}\right)$ $s_{sat}^{\alpha }\left(n_{1},v_{p},l_{c}\right)$ and $s_{cross}^{\alpha }\left(n_{1},v_{p},l_{c}\right)$ are represented as a functional forms in terms of the microstructural morphology. The functional forms are derived through the homogenization procedure. Therefore, a large number of sub-grain RVE model simulations with varying volume fractions, channel widths and shapes is generated and simulated in the dislocation density sub-grain scale where an explicit representation of the *γ*-*γ′* morphology is assigned in the RVE. For each morphology in the sub-grain scale, simulations in the homogenized single-crystal level are performed in order to satisfy macro-homogeneity [35,36]:

(17) $$\langle S\rangle :\langle \dot{E}\rangle =\frac{1}{\Omega_{RVE}}\int_{\Omega_{RVE}}S\;dV:\frac{1}{\Omega_{RVE}}\int_{\Omega_{RVE}}\dot{E}\;dV=\frac{1}{\Omega_{RVE}}\int_{\Omega_{RVE}}S:\dot{E}\;dV=\langle S:\dot{E}\rangle$$

Here, *S* and $\dot{E}$ correspond to the second Piola–Kirchhoff stress and the Lagrangian strain rate, respectively, and the symbol 〈*X*〉 corresponds to volume averaging over the RVE domain.

This extensive set of simulations, as explained in detail in [2], results in the following functional forms of the single-crystal constitutive parameters by using the least square minimization method in order to find the coefficients. The final form of these four constitutive parameters is as follows:

(18) $$\;s_{*0}^{\alpha }\left(n_{1},v_{p},l_{c}\right)=a_{1}\left(n_{1},v_{p}\right)+\frac{b_{1}\left(n_{1},v_{p}\right)}{\sqrt{l_{c}}}=-50v_{p}n_{1}+222v_{p}-34n_{1}+384+\;\;\frac{-33.3v_{p}n_{1}+32.92v_{p}+19.61n_{1}-0.037}{\sqrt{I_{c}}}\;s_{\text{sat}}^{\alpha }\left(n_{1},v_{p},l_{c}\right)=a_{2}\left(n_{1},v_{p}\right)+\frac{b_{2}\left(n_{1},v_{p}\right)}{l_{c_{1}}}=6680v_{p}n_{1}-8905v_{p}-1648n_{1}+3185+\;\;\frac{-3359v_{p}n_{1}+5008v_{p}+3631n_{1}-0.21}{\sqrt{I_{c}}}\;\;k_{*}\left(n_{1},v_{p},l_{c}\right)=19847v_{p}n_{1}l_{c}+12768v_{p}n_{1}-23120v_{p}l_{c}\;\;+4080n_{1}l_{c}-7500v_{p}+33n_{1}-2700l_{c}+65\;k\left(n_{1},v_{p},l_{c}\right)=a_{3}\left(n_{1},v_{p}\right)+\frac{b_{3}\left(n_{1},v_{p}\right)}{\sqrt{l_{c}}}=221.4v_{p}n_{1}-327.6v_{p}+31.5n_{1}+5.5+\;\;\frac{-176.5v_{p}n_{1}+281.2v_{p}-2.44n_{1}+0.14}{\sqrt{I_{c}}}\;\;s_{\mathrm{cross}}^{\alpha }\left(n_{1},v_{p},l_{c}\right)=s_{\mathrm{cross}}^{\alpha }*s_{0}\left(n_{1},v_{p},l_{c}\right),s_{0}\left(n_{1},v_{p},l_{c}\right)=a_{1}\left(n_{1},v_{p}\right)+\frac{b_{1}\left(n_{1},v_{p}\right)}{\sqrt{l_{c}}}\;\;=-50.32v_{p}n_{1}+0.538v_{p}-0.09528n_{1}+1+\;\;\frac{-0.08662v_{p}n_{1}+0.08566v_{p}+0.051n_{1}-0.000096}{\sqrt{l_{c}}}$$

The size-dependent variable, the channel width *l*_*c*_, can be seen in the parameters to reflect explicitly the size effect due to the presence of geometrically-necessary dislocations in the sub-grain scale of the model. In equation (18), the unit of *l*_*c*_ is *μ*m, while the units of initial thermal resistance and saturation shear resistance are MPa, and *s*_0_ is a dimensionless function.

### 4.4. Validation of the Homogenized Single-Crystal Grain-Scale Model

**Figure 5.**
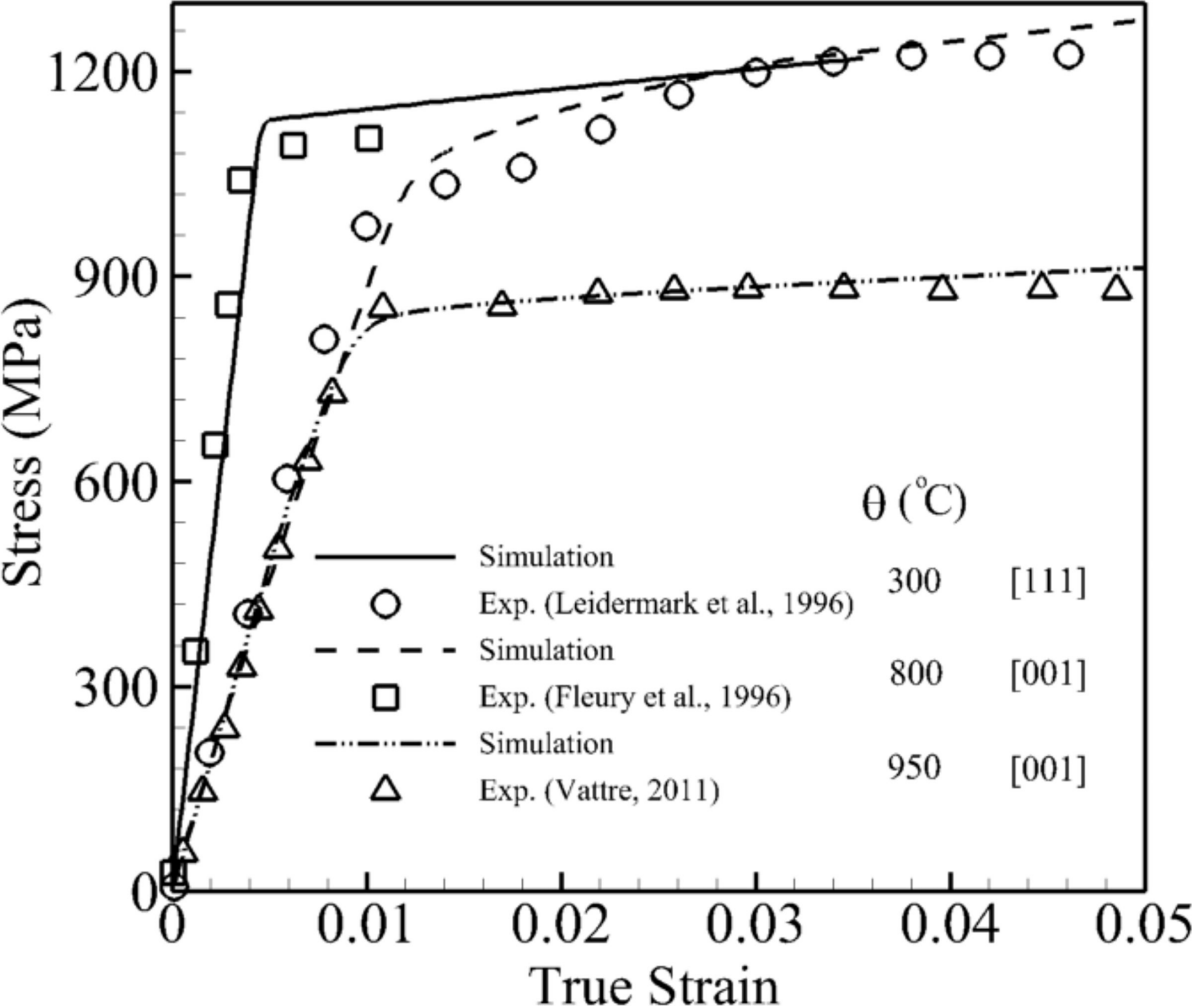
Volume-averaged true stress-logarithmic strain response by CPFEM and experiments [28–30] under a constant strain rate 0.001 s^−1^.

In order to validate the parametric constitutive model at the single-crystal grain scale, tensile constant strain tests are simulated for two orientations [1] and [111] for three temperatures 25 °C [29], 800 °C [28] and 950 °C [30] shown in Figure 5. The simulation at room temperature is performed for the [111] orientation and shows high yield stress because cubic slip systems are not activated at this temperature. Furthermore, the transition from the elastic to plastic part is very sharp, which shows that dislocation activities in the channel and matrix start almost simultaneously. Two simulations under elevated temperature are done for [1] orientation. In both simulations, it can be observed that initially plastic deformation starts in the channel where the slope of the elastic part changes for the stress around 600 MPa. However, this change is not significant due to the very small volume fraction of the channel. The yield stress and hardening decrease dramatically from 800 °C–950 °C. The simulations show a very good agreement with the experimental data.

## 5. Morphology Effect in Nickel-Based Superalloys

**Figure 6.**
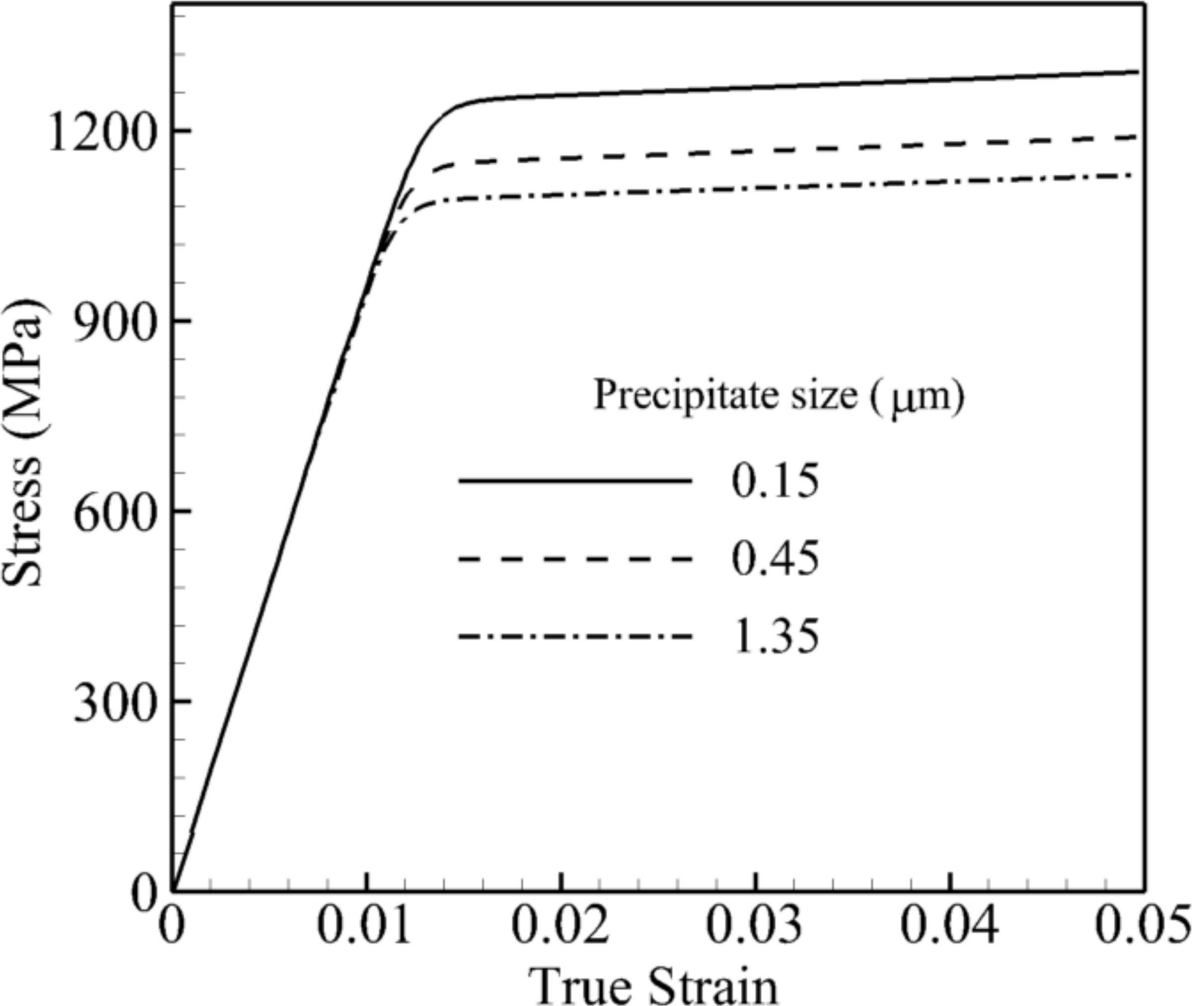
Volume-averaged true stress-logarithmic strain response by CPFEM under the strain rate of a single crystal of a nickel-based superalloy to investigate the effect of precipitate size at 800 °C under a tensile strain rate of 0.001 s^−1^.

So far, all simulations are performed for CMSX-4, which is a single crystal of a nickel-based superalloy containing 70% of cuboidal precipitates with an average distance of 0.45 μm. The main idea of this work is to bridge the morphology effect from an explicit representation (sub-grain scale) to an implicit one (grain scale) in order to get the same response with significant savings in time and computation. Therefore, the multi-scale scheme could greatly benefit the design and optimization [37–39] for the next generation of these alloys. In this section, the effect of morphology on the mechanical behavior of these alloys is investigated as a function of three independent morphology parameters: precipitate size, volume fraction and shape. In the first set of simulations, the size of the precipitates is changed from a very small particle size (0.15 μm) to a large one of (1.35 μm), while the volume fraction and the shape of precipitates are kept constant at 70% and *n* → ∞, respectively. In other words, for a unit cube of this material for one particle of *γ′* of a dimension of 1.35 μm, there will be 27 particles of 0.45 μm and 729 particles of a dimension 0.15 μm. The stress-strain curve under a tensile constant strain rate of 0.001 s^−1^ and 800 °C for these three sizes is shown in Figure 6. There is almost a 200-MPa difference between the 0.15 μm and 1.35 μm sizes. Smaller precipitate sizes for the same volume fraction and shape result in more particles, and more particles increase the dislocation densities and shear resistance.

**Figure 7.**
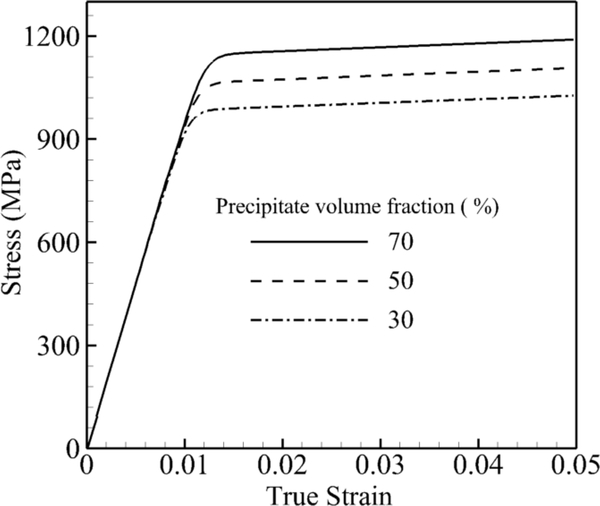
Volume-averaged true stress-logarithmic strain response by CPFEM under the strain rate of a single crystal of a nickel-based superalloy to investigate the effect of the precipitate volume fraction at 800 °C under a tensile strain rate of 0.001 s^−1^.

In the second set of simulations, the volume fraction of the precipitates is altered from a very low volume fraction of 30% to a high volume fraction of 70%, while the size and the shape of precipitates are kept constant at 0.45 μm and *n* → ∞, respectively. In other words, for a unit cube of this material, the channel width between precipitates in the case of 50% is 1.82-times and 30% is 2.95-times its width at a 70% volume fraction. The stress-strain curve under a tensile constant strain rate of 0.001 s^−1^ and 800 °C for these three volume fractions is shown in Figure 7. There is almost a 250-MPa difference between the 70% and 30% volume fractions. A larger volume fraction of the precipitate or a smaller channel width for the same size and shape increases the dislocation densities and shear resistance.

**Figure 8.**
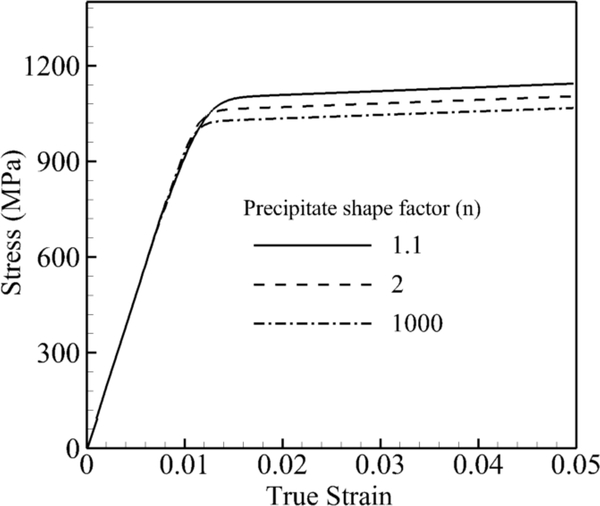
Volume-averaged true stress-logarithmic strain response by CPFEM under the strain rate of a single crystal of a nickel-based superalloy to investigate the effect of precipitate shape at 800 °C under a tensile strain rate of 0.001 s^−1^.

In the last set of simulations, the shape of the precipitates is changed from a small shape factor *n* = 1.1 to a large shape factor *n* = 1000, while the size and volume fraction of precipitates are kept constant at 0.45 μm and 70%, respectively. In other words, the surface boundary between precipitates is very smooth in the case of a smaller shape factor, and it is very sharp in the case of *n* = 1000, which looks like a cube. The stress-strain curve under a tensile constant strain rate of 0.001 s^−1^ at 800 °C for these three shape factors is shown in Figure 8. The difference between the lowest and highest yield stresses is not as pronounced as for the size and volume effects; however, the transition between the elastic and plastic part is sharper for the bigger shape factors. From the figure, the dislocation activities or plastic deformation begin at the stress around 700 MPa where three curves start to have a slight divergence. The slope of the curve from this point is higher for higher shape factors, which indicates less plastic deformation in the channel due to the sharp precipitate edge. Therefore, round-shaped precipitates increase the dislocation density due to more dislocation bowing around precipitates and result in bigger yield stress.

### 5.1. Flow Stress Variations with Morphology in Nickel-Based Superalloys

The yield strength corresponding to 0.2% offset strain is considered to investigate the dependencies on the morphology of nickel-based superalloys. Accordingly, crystal plasticity simulations are performed for different morphologies including different shapes, volume fractions and sizes of precipitates.

#### 5.1.1. Flow Stress Variations with the Shape of Precipitates

**Figure 9.**
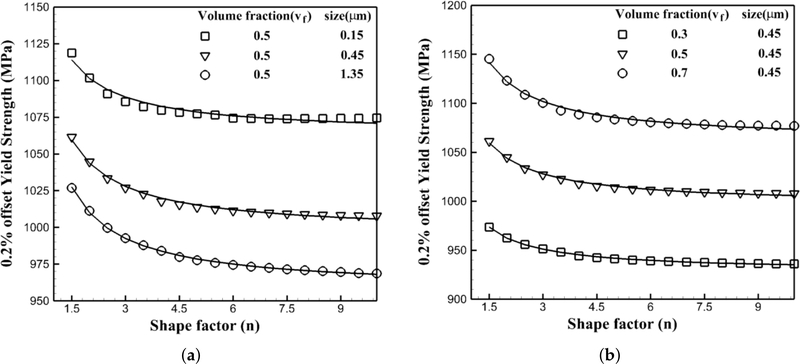
Variations of the 0.2% yield strength of a single crystal of a nickel-based superalloy with respect to the shape of precipitates at 1000 K under a tensile strain rate of 0.001 s^−1^: (**a**) constant volume fraction for different sizes of precipitates; (**b**) different volume fractions for a constant size of precipitate.

Crystal plasticity finite element simulations are performed at 1000 K under a strain rate of 0.001 s^−1^ for the crystal orientation [1] with the different shapes of precipitates while their volume fraction and size are constant for each set. In the first set of simulations, the volume fraction is constant and equal to 50%, while three sets of simulations are done for different sizes of precipitates, respectively 0.15 μm, 0.45 μm and 1.35 μm. In the second set, the size is fixed and equal to 0.45 μm, while the volume fraction changes as 30%, 50% and 70%, respectively. The results of the CPFEM simulations are shown in Figure 9 with symbols. The solid lines are plotted as the best trending functions to present these data. For all six plots, the functional form of $a+\frac{b}{n}$ is the best trending function to represent the data where *n* is the shape factor of precipitates defined in Section 4.1. In the the first set shown in Figure 9a, *a* and *b* are functions of the size of precipitates as *a*(*r*) and *b*(*r*), where in the second set shown in Figure 9b, they are functions of the volume fraction of the precipitates as $a\left(v_{f}\right)$ and $b\left(v_{f}\right)$. Hence, the flow stress in nickel-based superalloys will change proportionally with the shape factor of the morphology as 1/*n*.

#### 5.1.2. Flow Stress Variations with the Size of Precipitates

**Figure 10.**
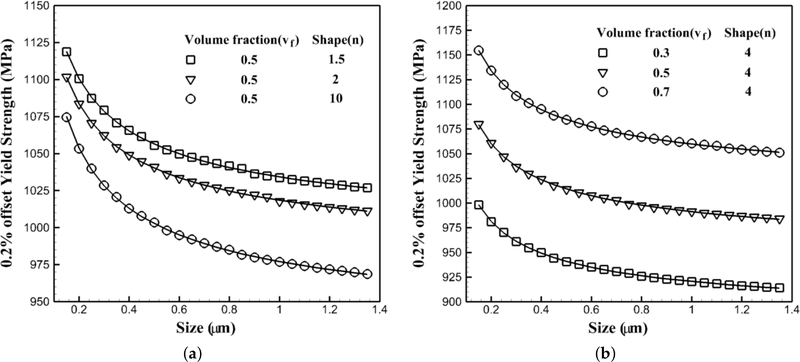
Variations of the 0.2% yield strength of a single crystal of a nickel-based superalloy with respect to the size of precipitates at 1000 K under a tensile strain rate of 0.001 s^−1^: (**a**) constant volume fraction for different shape of precipitates (**b**) different volume fractions for a constant shape of precipitate.

Crystal plasticity finite element simulations are performed at 1000 K under a strain rate of 0.001 s^−1^ for the crystal orientation [1] with the different sizes of precipitates, while their shapes and volume fractions are constant for each set. In the first set of simulations, the volume fraction is constant and equal to 50%, while three sets of simulations are done for different shapes of precipitates: 1.5, 2 and 10, respectively. It should be mentioned that the shape factor of two corresponds to the spherical shape for precipitates, while 10 represents almost a cubic shape of the precipitates. In the second set, the shape factor is fixed and equal to four, while the volume fraction changes as 30%, 50% and 70%, respectively. The results of the CPFEM simulations are shown in Figure 10 with symbols. The plotted solid lines are the best trending functions to present CPFEM data. For all six plots, the functional form of $a+\frac{b}{\sqrt{r}}$ is the best trending function to represent the data where *r* is the size of precipitates. In the the first set shown in Figure 10a, *a* and *b* are functions of the shape factor of precipitates as *a*(*n*) and *b*(*n*), where in the second set shown in Figure 10b, they are functions of the volume fraction of the precipitates as $a\left(v_{f}\right)$ and $b\left(v_{f}\right)$. Hence, the flow stress in nickel-based superalloys will change proportionally with the size of the morphology as $1/\sqrt{r}$.

#### 5.1.3. Flow Stress Variations with the Volume Fraction of Precipitates

**Figure 11.**
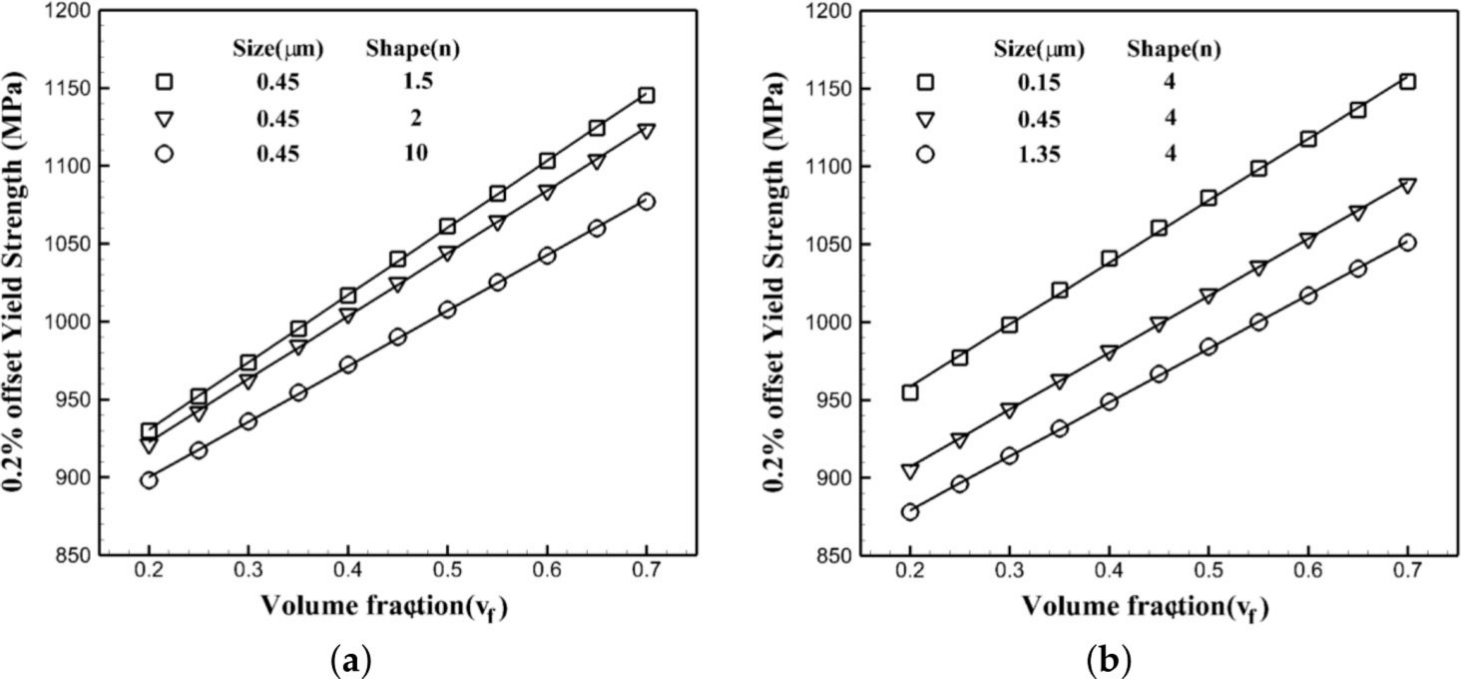
Variations of the 0.2% yield strength of a single crystal of a nickel-based superalloy with respect to the volume fraction of precipitates at 1000 K under a tensile strain rate of 0.001 s^−1^: (**a**) constant size for different shapes of precipitates; (**b**) different sizes for a constant shape of precipitate.

Crystal plasticity finite element simulations are performed at 1000 K under a strain rate of 0.001 s^−1^ for the crystal orientation [1] with the different volume fraction of precipitates while their shape and size are constant for each set. In the first set of simulations, the size is constant and equal to 50%, while three sets of simulations are done for different shapes of precipitates: 1.5, 2 and 10, respectively. In the second set, the shape factor is fixed and equal to four, while the size of precipitates changes as 0.15 μm, 0.45 μm and 1.35 μm, respectively. The results of the CPFEM simulations are shown in Figure 11 with symbols. The solid plotted lines are the best trending functions to present these data. For all six plots, the functional form of *a*+*bv*_*f*_ is the best trending function to represent the data where *v*_*f*_ is the volume fraction of precipitates defined in Section 4.1. In the the first set shown in Figure 11a, *a* and *b* are functions of the shape of precipitates as *a*(*n*) and *b*(*n*), where in the second set shown in Figure 11b, they are functions of the size of the precipitates as *a*(*r*) and *b*(*r*). Thus, the flow stress in nickel-based superalloys will change linearly with the volume fraction of the morphology as *v*_*f*_.

#### 5.1.4. Functional Form of the Flow Stress with Respect to the Morphology of Precipitates

As presented in the last section, the flow stress changes by changing the morphology of the microstructure. The change is proportional with 1/*n* with the shape of the precipitates while it changes as $1/\sqrt{r}$ with the size of the precipitates and linearly with the volume fraction of the precipitates. Therefore, the flow stress in the single crystals of nickel-based superalloys can be stated as:

(19) $$\sigma_{y}=\left(c_{1}+\frac{c_{2}}{n}\right)\left(c_{3}+\frac{c_{4}}{\sqrt{r}}\right)\left(c_{5}+c_{6}v_{f}\right)$$

According to this equation, the size of precipitates has the major effect on the yield stress, where the behavior shows a sort of Hall–Petch effect. The Hall–Petch effect represents the variation of the yield stress with respect to the size of the grain in a polycrystalline microstructure. The three variables in this equation, the shape, size and volume fraction of the precipitates, affect the channel width between precipitates. While the channel width between precipitates becomes narrower, the dislocation will have a difficult time passing through the channel. The size of precipitates in the nickel-based superalloys is related to the distance between precipitates for a specific shape and volume fraction. For example, if we have a cubic two-phase *γ*-*γ′* microstructure with the unit dimension, the average size of precipitates would be 0.8 of the unit for the volume fraction of 51.2% with cubic precipitates. In order to change the size of precipitates to 0.4 units while the volume fraction is 51.2% and the shape of the precipitates is cubic, we have to change the size of the microstructure; therefore, the size of the cubic microstructure becomes half of a unit. When the size of the microstructure decreases, the precipitates become closer, and the channel width becomes narrower. Hence, for a specific volume fraction and shape, finer precipitates mean less size in the microstructure, which results in less distance between precipitates or less channel width. Dislocations have a difficult time passing through the channel with less distance between precipitates, so they start to bow around the precipitates and result in the generation of another source of hardening, which is geometrically-necessary dislocations (GNDs). The generation of these dislocations accordingly results in increasing the yield stress. The decrease in the channel width, when the shape of precipitates changes, is not as sharp as changing the size of precipitates; therefore, the increment in the yield stress for lesser shape factor or more curvy precipitates is not the same as the increment seen in the size; however, the explanation stays the same. Dislocation will have more space to bow around precipitates and creates additional sources of dislocations to harden materials with a lesser shape factor. There will be the same explanation for increasing the yield stress by increasing the volume fraction. Having a greater volume fraction for the same size and shape of precipitates means less channel width between precipitates, which directly results in increasing GNDs.

Different optimization methods can be used in order to find the unknown coefficient. However, the least square method is employed in this work in order to calculate the constants *c*_1_–*c*_6_. The input date are obtained from 324 crystal plasticity finite element simulations, which are performed at 1000 K under a tensile strain rate of 0.001 s^−1^ as shown in Figures 9–11 and used for the least square method as the input data. The functional form of the flow stress as a function of shape, size and volume fraction of precipitates is calculated as:

(20) $$\sigma_{y}=1069.88-345.39v_{f}+\frac{34.67}{n}+\frac{181.14v_{f}}{n}-\frac{146.97}{\sqrt{r}}+\frac{425.51v_{f}}{\sqrt{r}}-\frac{19.15}{n\sqrt{r}}-\frac{0.411v_{f}}{n\sqrt{r}}$$

In this equation, the volume fraction of precipitates can change from 0.2–0.7; the shape factor of precipitates can change from 1.5–10; and the size of precipitates can vary from 0.15 μm–1.35 μm. The result of the above equation will be the flow stress in MPa.

#### 5.1.5. Validation of the Functional Form of Flow Stress with Respect to the Morphology of Precipitates

In order to validate the functional form of the flow stress given in Equation (20), 10 random morphologies are created and first simulated with the crystal plasticity finite element model, then the results are compared with the ones obtained from Equation (20). All simulations are performed at 1000 K under a tensile strain rate of 0.001 s^−1^. The results and comparison are shown in Table 6. As can be seen, the functional form of the flow stress obtained from the multi-scale framework gives almost identical flow stress compared with the crystal plasticity finite element models.

## 6. Conclusions

This paper proposes a functional form for the flow stress of single crystals of nickel-based superalloys as a result of a multi-scale crystal plasticity finite element framework. The multi-scale scheme bridges two scales in a hierarchical framework from the two-phase *γ*-*γ′* sub-grain scale to a homogenized single-crystal grain-scale constitutive model that can be augmented to model polycrystalline microstructures of nickel superalloys. The non-Schmid constitutive models for two scales include the main dislocation mechanism active in these materials. For the single-crystal grain scale, an activation-energy-based crystal plasticity finite element model is developed that incorporates non-Schmid effects along with the characteristic parameters of the sub-grain scale *γ*-*γ′* morphology. For the next scale, a crystal plasticity homogenized model is used, which includes the effect of the morphology implicitly through the homogenized constitutive parameters. A notable advantage of this multi-scale model is that its high efficiency enables it to be effectively incorporated in the polycrystalline grain scale of crystal plasticity finite element simulations, while retaining the accuracy of detailed RVE models. The homogenized model incorporates the effect of important characteristics of the sub-grain *γ*-*γ′* morphology, viz. the size, shape and volume fraction of the precipitates. The uniformly-distributed precipitates in the sub-grain RVEs in the shape of generalized ellipsoidal particles provide a platform for a modeling framework connecting the two scales, one with explicit representation and the other with their respective parametric forms. There is a size dependency in the two-scale model, where it naturally occurs in the sub-grain scale due to the presence of geometrically-necessary dislocations or GNDs, and it is reflected in the homogenized single-crystal grain-scale model through the explicit dependence on the channel width. The homogenized activation-energy-based crystal plasticity model is found to accurately reproduce the stress-strain response of the detailed RVE for a range of microstructural variations. It is also found to agree quite well with the results of experimental studies on single-crystal superalloys in the literature. The morphology parameters include the shape factor, volume fraction and size of the precipitates, which have different impacts on the flow stress. The functional form of the flow stress obtained from the multi-scale framework gives almost identical flow stress in comparison with the crystal plasticity finite element models.

## Figures and Tables

**Table 1.** Calibrated material constants for the yield state of the constitutive model according to the experimental data.

Parameter	*p_oct_*	*p_cub_*	*Q*(J)	*k*_1_	*k*_2_
Value	1.1	1.2	1.1 × 10^−20^	0.5	0.6

**Table 2.** Calibrated material constants for the temperature state of the constitutive model based on the experimental data.

Parameter	*ξ*_0_	*A*	*θ_c_*
Value	1.8	325	1400

**Table 3.** Calibrated material constants for the hardening state of the constitutive model based on experimental data.

Parameter	*f*_0_ (1/*s*)	*c*_0_ (1/*m*)	*c*_1_	*c*_2_	*c*_3_	*c*_4_	*c*_5_	*c*_6_	*c*_7_	*c*_8_
Value	10^8^	0.078	−3.77	4	0.3	100	0.001	0.0001	10	10

**Table 4.** Time integration scheme in CPFEM-MAT. APB, anti-phase boundary.

**A**. For time increment from *t* to *t* + Δ*t* with known **F**(*t* + Δ*t*) all known variables at time *t*
i. Calculate $S^{tr}=\frac{1}{2}C:(A(t+\Delta t)-I)$ using Equations (10) and (11).
ii. Evaluate the resolved shear stress due to trial stress $\tau^{\alpha }=S^{tr}:s_{0}^{\alpha }$, and update deformation variables in Step **B**.
iii. From Equation (10), calculate the first iterate $S^{1}(t+\Delta t)=S^{tr}-\sum_{\alpha =1}^{N}\Delta \gamma^{\alpha }(t+\Delta t)C^{\alpha }$
iv. For the *i*-th iteration in the Newton–Raphson method
(a) Evaluate $\tau^{i\alpha }=S^{i}(t+\Delta t):s_{0}^{\alpha }$, and update deformation variables in Step **B**.
(b) Using Equations (11) and (12), evaluate **S**^*i*+1^(*t* + Δ*t*) = **S**^*i*^(*t* + Δ*t*) – (**d**^*i*^)^−1^**G***^i^* where ${\left(d^{i}\right)}^{-1}=I+\sum_{\alpha =1}^{N}C^{\alpha }⊗\frac{\partial \Delta \gamma^{i\alpha }(t+\Delta t)}{\partial S^{i}(t+\Delta t)}$ and $G^{i}=S^{i}(t+\Delta t)-S^{tr}+\sum_{\alpha =1}^{N}\Delta \gamma^{\alpha }(t+\Delta t)C^{\alpha }$
(c) Verify convergence: If no, go to Step (a); if yes, go to Step v.
v. Evaluate $\tau^{(i+1)\alpha }=S^{i}(t+\Delta t):s_{0}^{\alpha }$, and update deformation variables in Step **B**.
vi. From Equation (9), evaluate $F^{p}(t+\Delta t)=\left(I+\sum_{\alpha =1}^{N}\Delta \gamma^{\alpha }(t+\Delta t)s_{0}^{\alpha }\right)F^{p}(t)$
vii. Calculate **F**^*e*^(*t* + Δ*t*) = **F**(*t* + Δ*t*)**F**^–*p*^(*t* + Δ*t*), $\sigma (t+\Delta t)=\frac{1}{det\left(F^{\mathrm{eT}}(t+\Delta t)\right)}F^{eT}(t+\Delta t)S(t+\Delta t)F^{e}(t+\Delta t)$ and $W=\frac{\partial \sigma }{\partial \varepsilon }$
**B**. Update deformation variables at any stage
I. Calculate dislocation density increments ${\dot{\rho }}_{SSD}^{\alpha },\rho_{CSD}^{\alpha },{\dot{\rho }}_{GNDs}^{\alpha },{\dot{\rho }}_{GNDet}^{\alpha }$ and $\rho_{GNDen}^{\alpha }$
II. Evaluate forest and parallel dislocation densities *ρ_P_*, *ρ_F_* and mobile dislocation density *ρ_m_* from Equation (5)
III. Check for the APB criterion given in Equation (6), then calculate cross-slip, passing and cutting shear resistances and the evolution of plastic shear strain from the Orowan equation by using Equation (3)

**Table 5.** Calibrated material constants for the single-crystal grain-scale activation-energy-based model.

Parameter	*k*_1_	*k*_2_	*ξ*_0_	*A*	*θ_c_*	*Q* (J)	*p*	*q*	$\dot{\gamma }\left(s^{-1}\right)$	*h*_0_ (MPa)	*r*
Value	0.4	0.6	8	325	1600	6.5 × 10^−19^	0.78	1.15	5 × 10^7^	100	1.115

**Table 6.** The CPFEM and Equation (20) results and comparison for the flow stress for different morphologies.

Shape Factor (*n*)	Volume Fraction (*v_f_*)	Size (*r*) (μm)	CPFEM Flow Stress (MPa)	Equation (20) (MPa)
1.7	0.31	0.45	973.6	976.9
2.2	0.32	0.37	975.1	969.3
2.5	0.36	0.43	980.6	983.2
2.3	0.44	0.57	1006.1	1009.8
2.9	0.48	0.55	1012.7	1014.3
3.5	0.52	0.31	1046.3	1050.6
4.2	0.57	0.38	1049.6	1053.4
6.7	0.61	0.45	1048.1	1044.4
7.3	0.41	0.37	986.9	984.0
8.1	0.44	0.31	1006.1	1000.1
